# Age-Specific Serum Anti-Mullerian Hormone and Follicle Stimulating Hormone Concentrations in Infertile Iranian Women

**DOI:** 10.22074/ijfs.2015.4205

**Published:** 2015-04-21

**Authors:** Alireza Raeissi, Alireza Torki, Ali Moradi, Seyed Mehdi Mousavipoor, Masoud Doosti Pirani

**Affiliations:** 1 Department of Biochemistry and Molecular Biology, Faculty of Medicine, Shahid Sadoughi University of Medical Sciences, Yazd, Iran; 2 Department of Biochemistry, International Campus of Shahid Sadoughi University of Medical Sciences, Yazd, Iran

**Keywords:** Anti-Müllerian Hormone, Follicle Stimulating Hormone, Infertility, Women, Age

## Abstract

**Background:**

Anti-Müllerian hormone (AMH) is secreted by the granulosa cells of growing follicles during the primary to large antral follicle stages. Abnormal levels of AMH and follicle stimulating hormone (FSH) may indicate a woman’s diminished ability or inability to conceive. Our aim is to investigate the changes in serum AMH and FSH concentrations at different age groups and its correlation with ovarian reserves in infertile women.

**Materials and Methods:**

This cross-sectional study analyzed serum AMH and FSH levels from 197 infertile women and 176 healthy controls, whose mean ages were 19-47 years. Sample collection was performed by random sampling and analyzed with SPSS version 16 software.

**Results:**

There were significantly lower mean serum AMH levels among infertile women compared to the control group. The mean AMH serum levels from different ages of infertile and control group (fertile women) decreased with increasing age. However, this reduction was greater in the infertile group. The mean FSH serum levels of infertile women were significantly higher than the control group. Mean serum FSH levels consistently increased with increasing age in infertile women; however mean luteinizing hormone (LH) levels were not consistent.

**Conclusion:**

We have observed increased FSH levels and decreased AMH levels with increasing age in women from 19 to 47 years of age. Assessments of AMH and FSH levels in combination with female age can help in predicting ovarian reserve in infertile women.

## Introduction

One of the main causes for infertility is decreased ovarian reserve. Ovarian reserve is the number of good quality oocytes that remain within the ovaries. As a woman’s age increases, her ovarian reserves decline (1,5). This decline may also result from autoimmune, genetic and iatrogenic conditions (6). In addition, autoimmune endocrinopathies, radiation therapy, or pelvic surgery may lead to decreased ovarian reserve (7,9). There are different tests to diagnose reductions in ovarian reserve such as elevated serum follicle stimulating hormone ( FSH ) levels on days 2 or 3 of the menstrual cycle FSH is mostly used in routine laboratories), low ovarian volume, an antral follicle count of <5 per ovary, low inhibin B levels, and <5 oocytes retrieved during an assisted reproductive technology ( ART ) cycle. A new test for the assessment of decreased ovarian reserve analyzes anti-Müllerian hormone ( AMH ) levels (10,14). AMH, a member of the transforming growth factor superfamily, is secreted in the human ovary by granulosa cells of primary growing follicles until the early antral stage (15,19). In females AMH regulates the growth of primary follicles by inhibiting further recruitment of other follicles during folliculogenesis (20). Serum AMH levels decline with increasing age in women and the levels are undetectable after menopause (21). Serum levels of AMH decrease prior to any increase in baseline FSH. FSH mostly indicates follicular maturation during the previous two weeks when gonadotropin follicles become sensitive. However AMH levels indicate pre-antral follicles to the post-primary pool that pass through stages before folliculogenesis (22,24). 

Few studies have assessed the levels of FSH and AMH at various ages in infertile women, therefore our aim was to investigate the changes in serum concentrations of AMH and FSH at different ages and its correlation with ovarian reserves in infertile women. 

## Materials and Methods

This cross-sectional study was performed at the Research Center of Infertility, Shahid Sadoughi University, Yazd, Iran between May 2010 and September 2012. We assessed serum AMH and FSH levels on days 2 or 3 of the menstrual cycles of 197 infertile women with problem decreasing ovarian reserve and 176 healthy controls, without decreasing ovarian reserve and age 19-47 years, who were admitted to infertility clinic to investigate infertility. Inclusion criteria were: no history of gynecological surgical procedures, presence of a regular menstrual cycle, no signs of hyper-androgenemia, and normal sonographic appearance of the ovaries. Infertile women were excluded if they were using fertility drugs or had any autoimmune, genetic, or iatrogenic conditions, autoimmune endocrinopathies, radiation therapy or pelvic surgery, or polycystic ovary syndrome as these factors have been shown to alter serum AMH levels (6,10). 

Patients were stratified into the following age categories: <25, 25-29.9, 30-34.9, 35-39.9, 40-45 and ≥45 years. Ethical approval for the study was received from the Women’s and Medical Ethics Committee. 

This approval allowed for measurement of serum AMH levels in stored routine clinical samples without the need for the patient’s written permission in order to produce an age-related normal range data for AMH levels. All 197 patients and 176 healthy controls were asked to provide their consent in order to link their AMH results with IVF outcome. 

Serum AMH levels were assessed in serum by the enzyme linked immunosorbent assay ( ELISA ) method ( Beckman Coulter, USA ). The sensitivity of the assay is 0.08 ng/ml with a reference range of 12.6 ng/ml. Interand intra-assay coefficients of variation ( CV ) are <7.7% and <5.8%, respectively. Luteinizing hormone ( LH ) and FSH levels were assessed in serum by the electrochemiluminescent immunoassay ( ECLIA ) method ( Cobas, England ). The assay sensitivity for FSH is <0.1 mIU/ml with interand intraassay CVs of <7.7% and <5.8%, respectively. Sample collection was performed by random sampling at days 3-5 of a spontaneous menstrual cycle. The serum was separated one hour after sampling and frozen at -20˚C until assayed. 

### Data analysis

The data were presented as mean±standard deviation as calculated in each group by SPSS software version 16 ( SPSS Inc., Chicago, IL, USA ). The student’s t test was used to assess differences between mean values of AMH, FSH and LH in the infertility and control groups with a confidence level of 95% and p value <0.05. 

## Results

**Fig.1:**
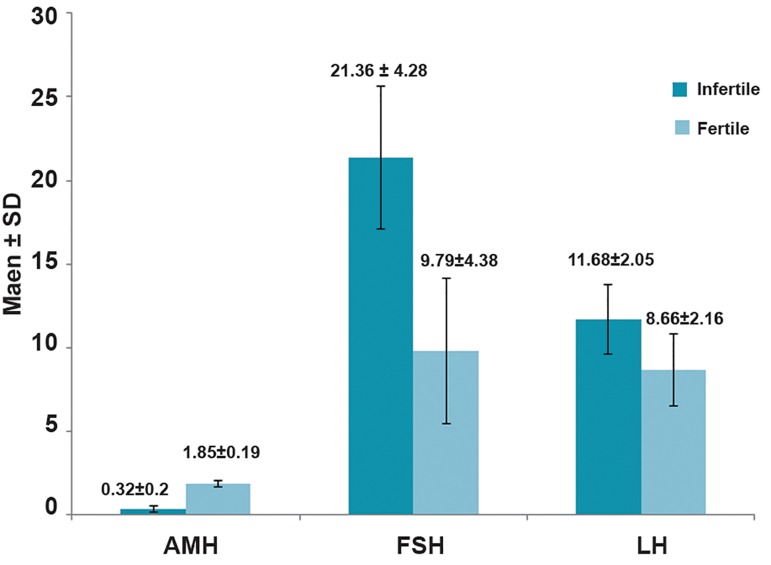
Mean±standard deviation in anti-Müllerian hormone (AMH), follicle stimulating hormone (FSH) and luteinizing hormone (LH) in both fertile and infertile women.

**Fig.2:**
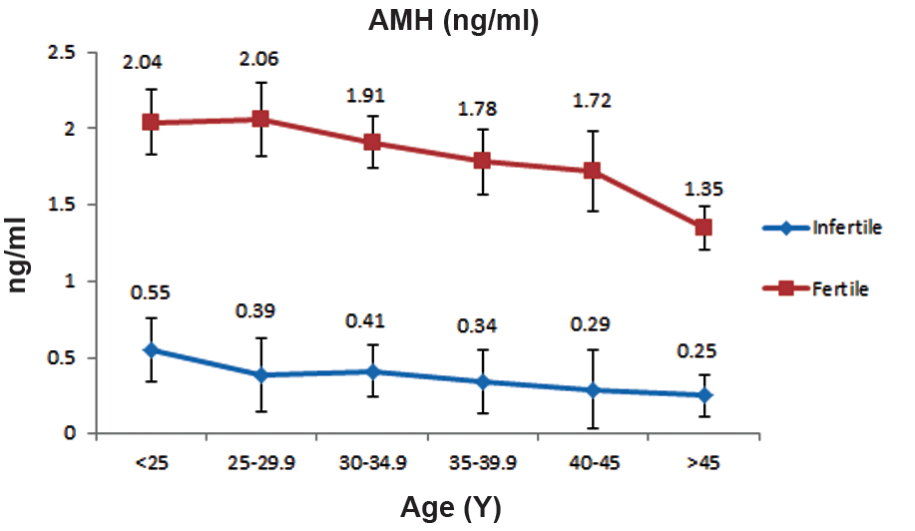
Mean±standard deviation values for anti-Müllerian hormone (AMH) over the reproductive age range in control group and infertile women.

Table 1 shows the mean AMH, FSH and LH levels according to the six age categories. Overall there was a significantly lower mean AMH serum level in infertile women compared to the control group (Fig.1,p=0.002). Although the mean serum AMH levels consistently decreased with increasing age in both the infertile and control groups, the reduction seen in the infertile group was more (Fig.2,p<0.05). Overall, the mean FSH serum level in infertile women was significantly higher than the control group (Fig.1,p=0.03). 

Mean serum FSH levels consistently increased with increasing age in both the infertile and control groups. 

**Fig.3:**
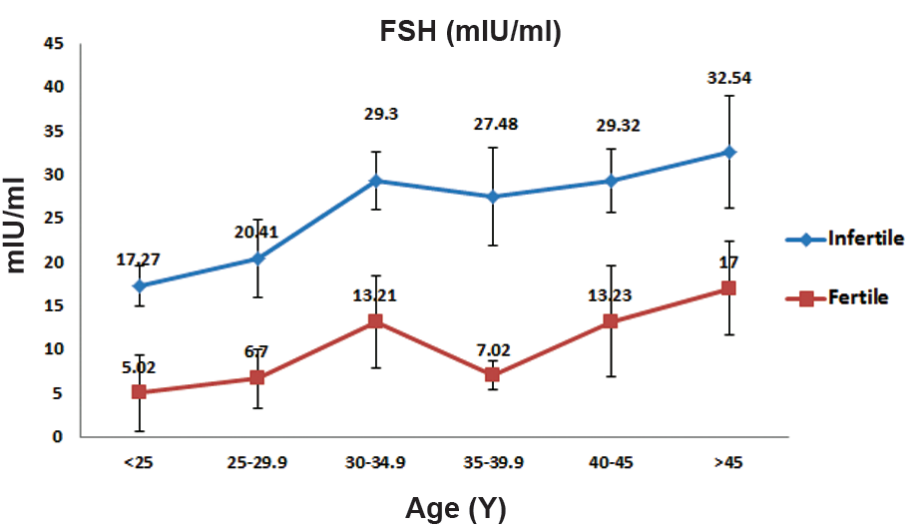
Mean±standard follicle stimulating hormone (FSH) values for the reproductive age range in control and infertile group women.

**Fig.4:**
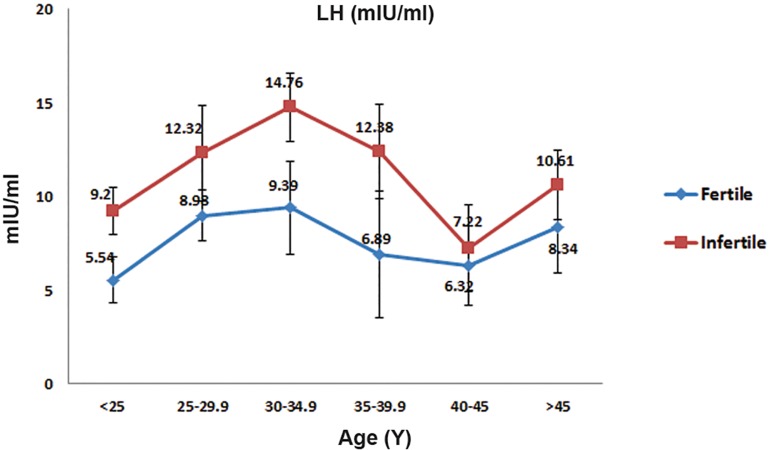
Mean±standard deviation values for follicle luteinizing stimulating hormone (LH) over the reproductive age range in fertile and infertile women.

However, this increase was higher in the infertile group (Fig.3,p<0.05). The mean LH serum level in infertile women was higher than the control group (Fig.1,p=0.32). The mean LH serum level in both groups with increasing age was not consistent (Fig.4). 

**Table 1:** Changes in anti-Mullerian hormone (AMH), follicle stimulating hormone (FSH) and luteinizing hormone (LH) serum concentrations for different ages of women in the infertile and control groups


Age (Y)		Infertile Mean±SD	Fertile Mean±SD	P value

		n=197	n=176	
**19-47 Total sample**	BMI (kg/m^2^)	27±2	27±6	
	AMH (ng/ml)	0.32±0.2	1.85±0.19	0.002
	FSH (mIU/ml)	21.36±4.28	9.79±4.38	0.03
	LH (mIU/ml)	11.68±2.05	8.66±2.16	0.32
		n=17	n=15	
**<25**	BMI (kg/m^2^)	28±3	27±5	
	AMH (ng/ml)	0.55±0.16	2.04±0.21	0.000
	FSH (mIU/ml)	17.27±2.36	5.02±4.39	0.002
	LH (mIU/ml)	9.2±1.25	5.54±1.23	0.2
		n=29	n=27	
**25-29.9**	BMI (kg/m^2^)	26±2	27±4	
	AMH (ng/ml)	0.39±0.23	2.06±0.24	0.001
	FSH (mIU/ml)	20.41±4.49	6.7±3.35	0.04
	LH (mIU/ml)	12.32±2.54	8.98±1.35	0.27
		n=38	n=32	
**30-34.9**	BMI (kg/m^2^)	28±1	26±7	
	AMH (ng/ml)	0.41±0.21	1.91±0.17	0.000
	FSH (mIU/ml)	29.3±3.24	13.21±5.26	0.02
	LH (mIU/ml)	14.76±1.81	9.39±2.49	0.35
		n=22	n=57	
**35-39.9**	BMI (kg/m^2^)	27±5	28±1	
	AMH (ng/ml)	0.34±0.18	1.78±0.21	0.000
	FSH (mIU/ml)	27.48±5.62	7.02±1.66	0.004
	LH (mIU/ml)	12.38±2.53	6.89±3.39	0.55
		n=61	n=31	
**40-44.9**	BMI (kg/m^2^)	28±6	26±3	
	AMH (ng/ml)	0.29±0.25	1.72±0.26	0.032
	FSH (mIU/ml)	29.32±3.59	13.23±6.34	0.023
	LH (mIU/ml)	7.22±2.3	6.32±2.12	0.34
		n=30	n=14	
**≥45**	BMI (kg/m^2^)	27±2	28±4	
	AMH (ng/ml)	0.25±0.22	1.35±0.14	0.012
	FSH (mIU/ml)	32.54±6.42	17±5.32	0.043
	LH (mIU/ml)	10.61±1.87	8.34±2.43	0.21


BMI; Body mass index.

## Discussion

In this study infertile women had higher FSH levels and lower AMH levels than fertile women. The range of AMH observed in infertile women was <1 whereas in the control group it was approximately 1 to 3. Mean serum AMH levels steadily decreased with increasing age in the age range of 19 to 47 years. In addition, mean FSH level approximately increased with increasing age in this range ( 19 to 47 years ) and was attributed to reduced ovarian reserve. Since AMH are produced by preantral and antral follicles (15,18,24,25), hence with increasing age, levels of pre-antral follicles decrease, causing a reduction in the amount of AMH. Thus, lower levels of AMH and higher FSH show declining ovarian reserve. Increased levels of FSH and decreased AMH can be considered as a marker for reduced fertility potential. This fluctuation on the third day FSH levels makes it difficult to predict ovarian reserve. Hence, the most appropriate factor for the assessment of ovarian reserve is an evaluation of AMH levels, which are independent of the cycle. 

We observed good correlation between serum AMH and FSH levels with ovarian reserve. These results supported those of previous studies in terms of the connections between low AMH serum levels and poor ovarian response (26,27). Several studies examined decreased AMH with age. Seifer et al. (25) reported that mean AMH levels decreased steadily with increasing age, in the range from 24 to 50 years, which was similar to the results of the current study. 

La Marca et al. (28), in a study on 277 healthy women ( aged 18-50 years ) reported that serum AMH levels progressively declined with increasing age. Mulders et al. evaluated serum AMH levels in 98 infertile and 48 healthy control women. They reported that serum AMH levels decreased over time in both infertile and healthy control women, which was similar to the results of our study (29). 

## Conclusion

With increasing age AMH levels decrease due to reduced ovarian reserves. Hence AMH can be used as a marker for the assessment of ovarian reserves in the follicular and luteal phases. 
